# Protein Quality and IgE-Binding in Ancient and Modern Wheat: An Analytical Study

**DOI:** 10.3390/foods15152605

**Published:** 2026-07-25

**Authors:** Dorota Piasecka-Kwiatkowska, Abhirami Remesan, Piotr Klimowicz, Ewa Springer

**Affiliations:** 1 Department of Food Biochemistry and Analysis, Poznan University of Life Sciences, 60-623 Poznan, Poland; abhiramirappu@gmail.com (A.R.); piotr.klimowicz@up.poznan.pl (P.K.); 2 Specialized Non-Public Health Care Facility Alergologia Plus, Allergy Diagnostics and Therapy Center, 60-693 Poznan, Poland; e.springer@wp.pl

**Keywords:** ancient wheat, protein quality, IgE-binding, gliadin, pasta processing, food allergy

## Abstract

Ancient wheat materials are increasingly used in cereal-based products because of their perceived nutritional advantages and distinctive protein composition. However, their protein quality and IgE-binding properties after processing remain insufficiently characterized. This study evaluated total protein content, essential amino acid composition, ELISA-detectable gliadin content, and IgE-binding patterns in flours and corresponding pasta products prepared from eight industry-sourced ancient and modern wheat materials, including einkorn, emmer, Kamut, spelt, round grain wheat, wheat 2Ab, common wheat, and durum wheat. Protein content was determined by the Kjeldahl method, amino acid composition by UHPLC, gliadin content by direct ELISA, and IgE-binding properties by slot blot analysis using sera from allergic individuals. The analysed wheat materials differed markedly in protein content, amino acid profile, gliadin detectability, and IgE-binding capacity. Spelt and Kamut showed the highest total protein contents, while selected ancient wheat materials, particularly emmer, Kamut, and einkorn, exhibited more favourable scores for some essential amino acids than common wheat and durum wheat. Nevertheless, none of the analysed materials fully met the FAO/WHO reference pattern for all evaluated essential amino acids, with lysine and histidine remaining the main limiting amino acids. Einkorn flour showed the highest ELISA-detectable gliadin content, consistent with its distinctive gluten protein composition. Processing into pasta reduced ELISA-detectable gliadin levels in all analysed materials, indicating processing-related changes in protein extractability and epitope accessibility. Despite reduced gliadin detectability after processing, IgE-reactive components remained detectable in both flour and pasta extracts. IgE-binding patterns were strongly dependent on wheat material, product form, and individual serum profile. Kamut pasta repeatedly showed one of the strongest IgE-binding responses across different sera, indicating that processing did not eliminate IgE-reactive components in this material. These findings demonstrate that selected ancient wheat materials may offer favourable protein-related nutritional characteristics, but these features should not be interpreted as evidence of reduced IgE-binding capacity. Integrated assessment of protein quality, gliadin detectability, and IgE-binding properties is therefore necessary for a more complete evaluation of ancient and modern wheat-based products.

## 1. Introduction

Wheat is one of the most important cereal crops worldwide and constitutes a major component of the human diet, supplying carbohydrates, proteins, dietary fiber, vitamins, minerals, and various bioactive compounds [1]. In addition, the technological importance in bread, pasta, and other cereal-based products, wheat proteins significantly contribute to human nutrition through the supply of essential amino acids and bioactive compounds [2]. However, wheat proteins are also associated with adverse immunological reactions, including wheat allergy, celiac disease, and non-celiac wheat sensitivity [3].

In recent years, ancient wheat species such as Einkorn (*Triticum monococcum*), Emmer (*Triticum dicoccum*), Kamut (*Triticum turgidum* ssp. *turanicum*), and Spelt (*Triticum spelta*) have attracted growing scientific and commercial interest because of their perceived nutritional advantages compared with modern wheat cultivars [4]. Several studies have reported that ancient wheats may contain higher protein contents, elevated mineral concentrations, and improved amino acid composition [4,5,6]. However, ancient wheats are not a uniform group, also because they differ in ploidy level and genome composition. Einkorn is diploid and carries the AA genome, emmer and Khorasan wheat/Kamut are tetraploid wheats with the AABB genome, while common wheat and spelt are hexaploid wheats with the AABBDD genome; durum wheat is also tetraploid. These differences are reflected, among others, in the composition and organization of storage proteins, especially gliadins and glutenins. Such differences in gluten protein composition among wheat genotypes have been associated with variation in technological properties, digestibility, analytical detectability, and immunological recognition [7,8,9]. Other recent findings have shown that ancient wheat species may also be characterized by higher contents of minerals and phenolic compounds, supporting their potential use as nutritionally valuable ingredients in cereal-based products aimed at health-conscious consumers [10]. Nevertheless, conflicting evidence remains regarding the allergenic potential of ancient wheats. While some studies demonstrate that ancient wheats can retain substantial IgE-binding activity comparable to or even exceeding that of modern cultivars [11].

Recent studies have further emphasized that the nutritional and functional properties of ancient wheat species may differ markedly among species and cultivars and can be substantially modified during germination and processing. In a recent study on several *Triticum* species, controlled germination altered storage protein fractions, including gliadins and glutenins, and was accompanied by changes in phenolic compounds and antioxidant activity [12]. These findings support the view that ancient wheats should not be considered a uniform group with consistently superior nutritional or functional properties. Instead, their potential application in cereal-based foods requires an integrated assessment of species, processing, nutritional quality, and immunological safety. From a nutritional perspective, protein content alone is not sufficient to define the quality of wheat-based products. The biological value of cereal proteins depends largely on their essential amino acid composition and on the presence of limiting amino acids, particularly lysine, which is typically deficient in wheat proteins [2,6]. Therefore, the assessment of ancient wheat species should include both quantitative protein determination and qualitative evaluation of amino acid profiles.

Gliadins and glutenins are the principal storage proteins in wheat and play a central role in both dough functionality and immunological responses [13]. Gliadins contribute mainly to dough viscosity and extensibility, whereas glutenins are responsible for elasticity and gluten network strength. Their relative proportions vary considerably among wheat species and cultivars, which may influence not only technological performance but also protein digestibility, analytical detectability, and immunological recognition [3,10]. Since gliadins include proteins recognized by antibodies used in gluten detection and by IgE from sensitized individuals, their quantification provides an important link between technological quality, analytical assessment, and immunological evaluation [1,11,13]. Processing conditions such as hydration, extrusion, heating, and drying may further modify protein structure, alter epitope accessibility, and influence allergen recognition. Pasta represents a relevant cereal product model because its production involves hydration, mixing, extrusion, drying, and subsequent cooking, all of which may modify gluten organization and protein. As a result, reduced detectability of gliadin after processing does not necessarily indicate the elimination of immunoreactive proteins [14,15]. Comparative analysis of flour and pasta is therefore important for understanding whether processing modifies nutritional quality and IgE-binding capacity in a species-dependent manner.

From an applied perspective, the evaluation of wheat materials actually used in food production is particularly relevant. Industrial pasta manufacturers use different wheat species and commercially available wheat materials to diversify product composition and nutritional value. Therefore, analyzing flours obtained directly from pasta production, together with the corresponding pasta products, provides practical insight into how raw material selection and processing may influence protein composition, nutritional quality, and analytical gliadin detectability.

Although several studies have separately investigated wheat protein composition, gliadin content, amino acid profile, or allergenicity, comparative studies integrating both nutritional and immunological evaluation of ancient and modern wheat species in flour and processed pasta products remain limited. Therefore, the present study aimed to evaluate protein quality and IgE-binding properties of flours and pasta products prepared from eight industry-sourced ancient and modern wheat materials. The study focused on materials relevant to industrial practice and consumer products, rather than on raw wheat kernels of defined cultivars. Protein quality was assessed based on total protein content and essential amino acid composition, while gliadin content was determined as an additional indicator of gluten protein composition and analytical detectability. The study further investigated whether industrial pasta processing modifies protein-related nutritional characteristics, gliadin detectability, and IgE-binding patterns using sera from allergic individuals. The findings provide insight into the relationship between wheat material, processing, protein quality, and IgE-binding capacity in wheat-based products.

## 2. Materials and Methods

### 2.1. Sample Collection and Pasta Preparation

Pasta samples were produced from modern wheat species, *Triticum durum* (durum wheat) and *Triticum aestivum* (common wheat), as well as from ancient wheat species, including *Triticum spelta* (spelt), *Triticum monococcum* (einkorn), *Triticum dicoccum* (emmer), and *Triticum turgidum* ssp. *turanicum* (Kamut), together with round grain wheat and 2Ab wheat. The corresponding whole-grain flours and pasta products were supplied by Makarony Polskie S.A. (Rzeszów, Poland).

All raw materials were sourced and processed under standardized industrial conditions to ensure traceable origin, uniform technological parameters, and consistent product quality. The analyzed flours and pasta samples, together with their respective identification codes, are presented in Table 1.

Eight pasta samples, each representing a different wheat species, were prepared for the immunoreactivity analysis. For each sample, 5 g of pasta was accurately weighed and transferred to a separate beaker. Subsequently, 50 mL of distilled water was added, and the samples were cooked under cover at 100 °C according to the manufacturer’s instructions for optimal al dente preparation. Most pasta samples were cooked for 6 min, whereas the common wheat pasta sample was cooked for 7 min, as indicated on the product packaging. This approach was used to ensure that each pasta sample reached its manufacturer-recommended preparation state before analysis. Gentle stirring was performed every 2 min to prevent agglomeration.

After cooking, the samples were cooled to room temperature and stored in airtight containers at 4 °C until further analysis.

Immunoreactivity (IgE-binding) was assessed using archived and fully anonymized human serum samples obtained from individuals treated at the Specialized Non-Public Health Care Facility (SNZOZ) Allergology Plus, Allergy Diagnosis and Therapy Center in Poznan, Poland. The serum samples originated from blood previously collected during routine allergy diagnostics and were not collected specifically for the purposes of the present study. The selected sera represented distinct sensitization profiles relevant to exploratory IgE-binding analysis, including sensitization to common inhalant allergens and, in selected cases, to wheat/flour or food allergens. The use of these sera was intended to assess whether IgE antibodies from individuals with different sensitization backgrounds recognize components present in wheat protein extracts. The sera used in the present study were archived and anonymized, and detailed clinical information on symptoms after ingestion of wheat, pasta, or other cereal products was not available for all patients. Therefore, the results should be interpreted as IgE-binding patterns of the tested extracts, rather than as evidence of clinically confirmed wheat allergy or clinically confirmed pollen-food cross-reactivity.

Each participant, or their legal representative, had provided written informed consent for the anonymous use of the previously collected residual serum samples in scientific research. No additional blood sampling, diagnostic procedure, therapeutic intervention, or contact with the participants was performed for the purposes of this study. Since the study used anonymized residual biological material that had been collected previously during routine diagnostic procedures and was not collected for scientific purposes, the study did not constitute a medical experiment under the applicable Polish legislation and approval from the Bioethics Committee was not required.

Allergenic classification of the sera was determined using Polycheck^®^ diagnostic kits (Biocheck GmbH, Münster, Germany), which quantify specific IgE (sIgE) levels. The Polycheck^®^ inhalation and/or food panels were selected according to individual clinical histories. Wheat flour or flour mix was included only in some of the diagnostic panels used; therefore, sIgE results for wheat/flour were not available for all sera. Where included, the test provided information on IgE sensitization to wheat flour or flour mix, but did not distinguish between individual wheat species or cultivars. A cut-off value of 0.35 kU/L was used to define positive sensitization, with allergenic classes defined as follows: Class 0 (<0.35 kU/L), Class 1 (0.35–0.7 kU/L), Class 2 (0.7–3.5 kU/L), Class 3 (3.5–17.5 kU/L), Class 4 (17.5–50 kU/L), Class 5 (50–100 kU/L), and Class 6 (>100 kU/L). Prior to immunoassays, sera were diluted 1:20 in TBS containing 1% bovine serum albumin.

### 2.2. Total Protein Content Determination (Kjeldahl Method)

Total protein content of flour and pasta samples was determined by the Kjeldahl method according to ISO:20483:2013 [16]. Approximately 0.5 g of each sample (dry weight basis) was digested in concentrated sulfuric acid in the presence of a selenium catalyst until complete mineralization. After cooling, the digests were subjected to ammonia distillation and titration. Nitrogen content was calculated from the titration values after blank correction and converted to protein using a nitrogen-to-protein conversion factor of 5.7, appropriate for wheat-based products. Dry matter content was determined before Kjeldahl analysis, and total protein values were calculated and expressed as g protein per 100 g dry matter. All analyses were performed in triplicate.

### 2.3. Amino Acid Profiling

Quantitative and qualitative amino acid profile analysis was performed using the UHPLC method. Two hydrolysis procedures were performed according to AOAC method 994.12-1997: acidic hydrolysis (110 °C, 23 h) and oxidative hydrolysis (4 °C, 16 h, and 100 °C, 2 h). The use of two separate analyses allowed the determination of 19 of the 20 protein amino acids: L-alanine (Ala), L-arginine (Arg), L-aspartic acid (Asp) + L-asparagine (Asn), L-glutamic acid (Glu) + L-glutamine (Gln), L-leucine (Leu), L-lysine (Lys), L-serine (Ser), L-threonine (Thr), L-tyrosine (Tyr), L-valine (Val), L-histidine (His), L-Isoleucine (Ile), L-Phenylalanine (Phe), L-Proline (Pro), Glycine (Gly) and two sulfur amino acids: L-methionine (Met) and L-cysteine (Cys). To detect amino acids using the PDA detector, samples were derivatized with AccQ•Tag reagents, (No. 186003836, Waters Corporation, Milford, MA, USA ) according to the manufacturer’s protocol and analyzed using a UHPLC chromatograph (Shimadzu Nexera 2.0, Kyoto, Japan). Separation was performed on an AccQ-Tag Ultra C18 column, 1.7 µm, 2.1 × 100 mm (Waters). The column temperature was 55 °C, the flow rate was 0.6 mL/min, and a non-linear separation gradient was created by mixing AccQ•Tag Ultra Eluent A and Eluent B (Waters). Detection was performed at 260 nm. To ensure standardized presentation of the amino acid profile independent of varying moisture or non-protein components, amino acids contents were expressed as g per 16 g N, equivalent to g per 100 g protein [17]. Tryptophan was not determined because it is degraded under the hydrolysis conditions used.

### 2.4. Gliadin Extraction and Quantification by ELISA

Gliadin-containing wheat protein extracts were prepared according to a previously described method [18]. Briefly, flour samples (0.5 g) were extracted with 5 mL of 70% (*v*/*v*) ethanol, vortexed for 5 min, and centrifuged. The supernatants were collected and analysed shortly after preparation or, when immediate analysis was not possible, stored in the dark at room temperature for no longer than 5 days in the present study, although the referenced method allows storage for up to 14 days. Prior to analysis, extracts were diluted 1:50 in Tris-buffered saline (TBS).

Gliadin content was determined using a direct ELISA. Microtiter plates were activated with carbonate–bicarbonate buffer (pH 9.6) and coated with gliadin extracts or gliadin standards (Sigma-Aldrich, G3375, St. Louis, MO, USA; 20–0.156 µg/mL), followed by incubation at 37 °C. After washing, plates were blocked with 1% bovine serum albumin in TBS-Tween and incubated with an anti-gliadin (wheat) peroxidase-conjugated polyclonal antibody (Sigma-Aldrich, A1052) diluted in TBS 1:1500. Because a polyclonal anti-gliadin antibody was used, the ELISA results were interpreted as gliadin content determined under the applied immunochemical assay conditions. Possible recognition of structurally related gluten protein components cannot be completely excluded. Color development was performed using o-phenylenediamine dihydrochloride (OPD, Sigma-Aldrich P9029, St. Louis, MO, USA) during a 15 min of incubation in the dark. The reaction was stopped with sulfuric acid, and absorbance was measured at 490 nm using Biochrom Asys UVM 340 Microplate (Biochrom, Cambridge, UK) microplate reader.

### 2.5. Detection of Immunoreactivity by Slot Blot

Protein slot blotting was performed using a Slot Blotter apparatus (Geneflow, Lichfield, UK), whereas the assay was performed using a SNAP i.d. 2.0 Protein Detection System (Merck iKGaA, Darmstadt, Germany), following the manufacturer’s instructions. An Immobilon-P PVDF membrane (Merck, Darmstadt, Germany; pore size: 0.45 µm, catalogue number: IPVH00010) was pre-activated according to the manufacturer’s recommendations and placed on the gasket of the apparatus. An equal volume of each wheat protein extract (200 µL) was applied to the PVDF membrane under vacuum, allowing protein immobilization. After the sample application, the wells were washed with Tris-buffered saline (TBS, pH 7.4) to remove unbound material. The membrane was then blocked with TBS containing 1% bovine serum albumin (TBS–1% BSA) for 30 min at room temperature with gentle agitation.

Following blocking, the membrane was incubated with patient serum (Table 2) diluted 1:20 in TBS–Tween buffer for 10 min at room temperature. After incubation, the membrane was washed three times with TBS–Tween buffer to remove unbound antibodies.

Subsequently, the membrane was incubated for 10 min with an anti-human IgE alkaline phosphatase-conjugated mouse monoclonal antibody (A3076, Sigma-Aldrich, Merck), diluted 1:2000 in TBS (pH 7.4) containing 1% BSA and 0.05% Tween 20. The membrane was then washed three times with TBS–Tween buffer.

For color development, BCIP/NBT substrate solution (5-bromo-4-chloro-3-indolyl phosphate/nitro blue tetrazolium, Calbiochem, San Diego, CA, USA) was applied, and the reaction was monitored until visible signal development. The reaction was stopped by rinsing with distilled water, and the membrane was air-dried prior to documentation. Densitometric analysis of protein bands was performed using a custom-developed software tool designed for the quantitative evaluation of signal intensity.

The slot blot assay was applied as a comparative immunochemical method to evaluate relative IgE-binding patterns of wheat protein extracts when probed with sera from allergic individuals. The assay was not intended to identify individual IgE-reactive protein fractions. Therefore, the observed IgE-binding signals should be interpreted as relative immunoreactivity of the tested extracts rather than as binding to purified gliadin alone. Because each membrane was processed independently and probed with a different patient serum, densitometric values were normalized and interpreted separately for each membrane/serum.

### 2.6. Statistical Analysis

Data were expressed as mean ± standard deviation of three replicates. Statistical analysis was performed using Statistica software (version 13; TIBCO Software Inc., Palo Alto, CA, USA). Differences among means were evaluated by analysis of variance (ANOVA), followed by Tukey’s post hoc test, with significance set at *p* < 0.05.

For all analysed parameters, statistical comparisons were performed separately within each product form. Thus, flour samples were compared with other flour samples, and pasta samples were compared with other pasta samples. Flour and pasta samples were not directly compared with each other by ANOVA, because the study was not designed as a mass-balance assessment of changes occurring during pasta processing, but rather as a comparative analysis of different wheat materials within each product form.

## 3. Results and Discussion

### 3.1. Protein Content in Wheat Species

**Figure 1:**
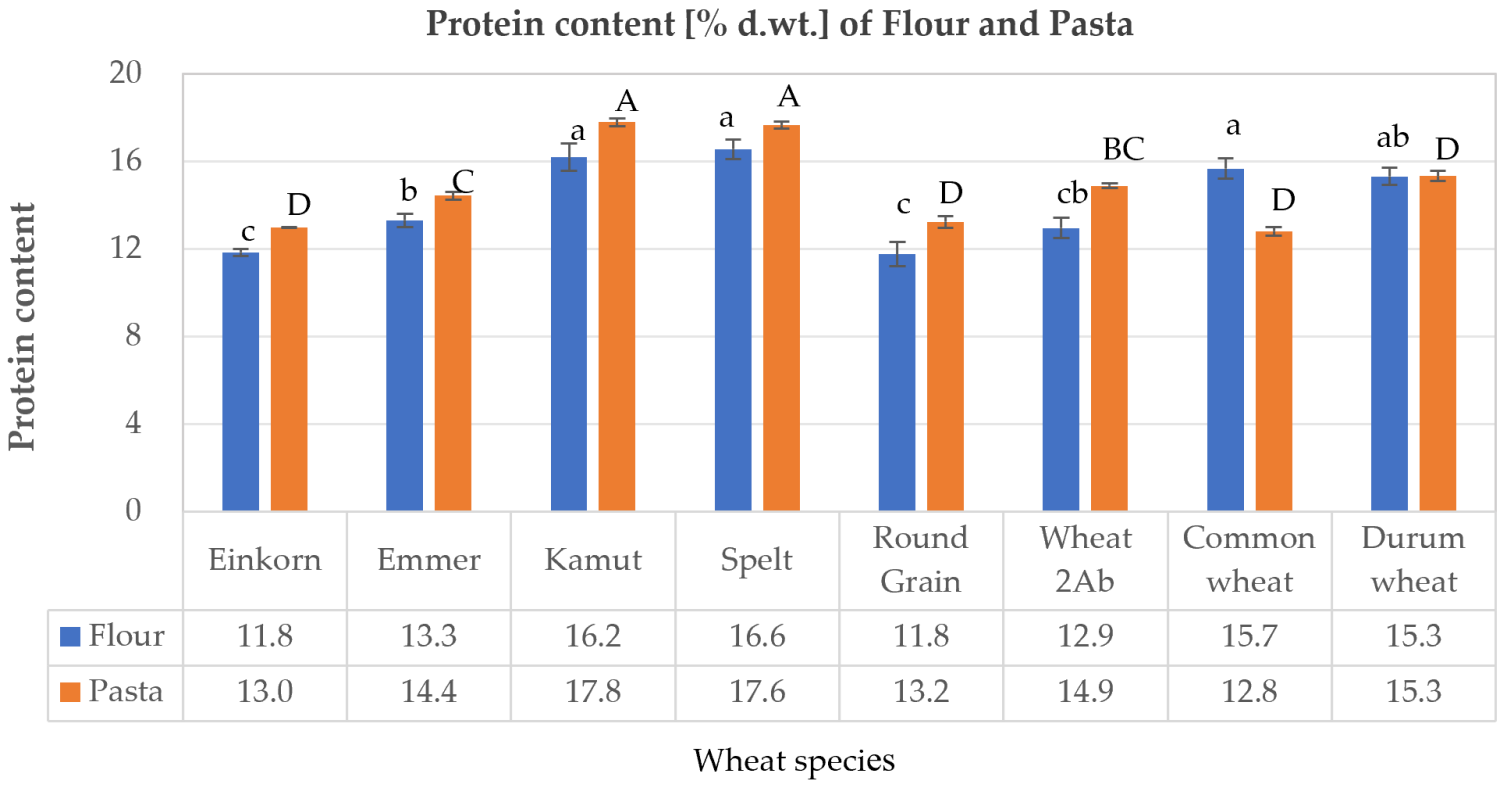
Protein content in wheat flour and corresponding pasta products determined by the Kjeldahl method and expressed as g protein per 100 g dry matter. Different lowercase letters indicate statistically significant differences among flour samples, whereas different uppercase letters indicate statistically significant differences among pasta samples according to Tukey’s HSD test (*p* < 0.05).

Protein content of flours and corresponding pasta products from different wheat species was determined using the Kjeldahl method (Figure 1). All values were expressed on a dry matter basis. Statistical comparisons were performed separately within flour samples and within pasta samples; therefore, flour–pasta pairs were not directly compared by ANOVA.

Significant differences were observed among flour samples. Spelt (16.6%) and Kamut (16.2%) showed the highest protein contents, followed by common wheat (15.7%), whereas einkorn and round grain wheat exhibited substantially lower values. Among pasta products, Kamut (17.8%) and spelt (17.6%) also had the highest protein contents, while common wheat, einkorn, and round grain wheat pastas showed lower values. These results indicate marked material-related differences in protein content within each product form. Because protein values were expressed on a dry matter basis, the observed flour–pasta differences cannot be attributed simply to moisture content. However, they should also not be interpreted as a direct increase or decrease in protein caused by pasta processing. The analysed samples were industry-sourced flours and corresponding final pasta products, and the study was not designed as a mass-balance experiment following protein recovery from a defined amount of flour into pasta. Therefore, flour and pasta data were used primarily to compare wheat materials within each product form, rather than to quantify processing-induced changes in total protein.

The observed differences are consistent with previous reports showing considerable variation in protein content among ancient and modern wheat materials [6,19]. However, because the analysed flours were obtained as industry-sourced materials and detailed cultivar information was not available, these differences should be interpreted as material-related rather than cultivar-specific effects. Protein content in wheat grain is known to be influenced not only by genetic background, but also by environmental conditions, agronomic practices, grain composition, and processing history. Therefore, the relatively high protein contents observed in spelt and Kamut should be considered characteristic of the analysed materials rather than generalized to all ancient wheat species.

Total protein content alone does not adequately represent technological or nutritional quality. Functional performance depends on protein composition, including gliadin-to-glutenin ratios and degree of polymerization, which affect rheology, digestibility, and processing behavior [20]. Although einkorn showed lower total protein content in the present study, total protein content alone is insufficient to assess protein-related nutritional quality. Differences in protein composition and amino acid profile should also be considered, supporting the need to evaluate qualitative parameters alongside quantitative protein determination [6]. Therefore, quantitative protein determination should be complemented by qualitative assessment of amino acid composition, as discussed in the following section.

### 3.2. Amino Acid Profile of Flours and Pasta from Different Wheat Species

The essential amino acid composition of wheat flours and corresponding pasta products, expressed as mg/g protein and as amino acid scores relative to the FAO/WHO reference pattern, is presented in Table 3 and Table 4. The analysed amino acids included histidine, isoleucine, leucine, lysine, aromatic amino acids, threonine, and valine, whereas sulphur amino acids and tryptophan were not included in the evaluation.

In flour samples, lysine was the most limiting essential amino acid relative to the FAO/WHO reference pattern. The highest lysine content was observed in emmer flour (30.0 mg/g protein), corresponding to 62.5% of the reference requirement. Einkorn also showed a higher lysine score than common wheat and durum wheat, although it remained below the reference requirement. In contrast, common wheat and durum wheat flours exhibited markedly lower lysine values, corresponding to approximately 2% of the FAO/WHO reference pattern. These results confirm that, despite clear differences among the analysed wheat materials, lysine remains a major limiting amino acid in wheat proteins.

Histidine contents were also low across all flour samples, ranging from 1.87% to 6.25% of the FAO/WHO reference pattern. The lowest histidine scores were observed in common wheat and durum wheat. Although lysine is generally considered the principal limiting amino acid in cereal proteins, the consistently low histidine values observed in the present study indicate that histidine may also contribute to the reduced nutritional score of the analysed wheat materials. Previous studies on bread wheat varieties from Serbian breeding centres in Novi Sad and Kragujevac reported variability in lysine and histidine contents among wheat materials [2]. Although the absolute values reported in that study are not directly comparable with the present results, they support the view that essential amino acid profiles in wheat may differ depending on the analysed material, genetic background, and environmental conditions.

Among the remaining essential amino acids, emmer and Kamut generally showed more favourable profiles than common wheat and durum wheat. Kamut flour exhibited the highest threonine score, whereas emmer flour showed relatively higher leucine, valine, and aromatic amino acid contents. The comparatively higher lysine scores observed in emmer and einkorn, compared with common wheat and durum wheat, indicate material-related differences in protein quality. This is consistent with previous studies showing that ancient wheat species, particularly einkorn, may exhibit distinctive protein and amino acid characteristics [6,21]. However, none of the analysed flour samples met the FAO/WHO reference pattern for all essential amino acids, indicating that ancient wheat materials should not be considered nutritionally superior as a uniform group.

The amino acid profiles of pasta products partly differed from those observed in the corresponding flours. As in the flour samples, lysine and histidine remained among the most limiting essential amino acids relative to the FAO/WHO reference pattern. Among the pasta products, Kamut pasta had the highest lysine score, whereas round-grain wheat pasta had the lowest lysine value. Histidine scores also remained low in most pasta samples, although higher values were observed in durum wheat and einkorn pasta compared with their corresponding flour samples.

For several essential amino acids, the measured values in pasta were higher than those observed in the corresponding flour samples. Since amino acid contents were expressed in relation to protein nitrogen, as g per 16 g N, these differences should not be interpreted as a direct increase in the absolute amount of amino acids during pasta processing. The data allow comparison of the analysed flour and pasta samples as product materials, because all samples were analysed using the same method and expressed in the same units. However, the study was not designed as a controlled mass-balance experiment following the same defined flour batch through pasta production. Moreover, raw wheat kernels were not analysed, and detailed information on cultivar composition, grain batches, milling yield, and grain-to-product mass balance was not available. Therefore, amino acid contents could not be reliably expressed per gram of initial raw wheat kernels. Flour–pasta differences in amino acid values were therefore interpreted as differences between the analysed product samples, expressed on a protein basis, rather than as evidence of processing-induced amino acid gains or losses.

At the same time, processing-related changes in the pasta matrix may contribute to the observed differences, including protein aggregation, starch–protein interactions, and altered analytical accessibility of protein fractions after hydration, extrusion, drying, and cooking. Previous studies have also shown that pasta processing and product formulation may influence essential amino acid composition and protein quality parameters in cereal-based products [22,23]. Therefore, these differences should be interpreted cautiously. Because amino acid values were expressed on a protein basis, they may be influenced by matrix effects and analytical recovery, they do not necessarily indicate a direct improvement in the intrinsic amino acid composition of wheat proteins or amino acid gains during pasta processing.

The material-related differences observed in the present study are consistent with previous reports showing that amino acid composition in wheat is influenced by wheat species, cultivar, genetic background, and growing conditions. Field studies have demonstrated variation in essential amino acids such as lysine, leucine, and valine, confirming that protein quality traits are shaped by both genetic and environmental factors [24]. In particular, einkorn has been described as a nutritionally distinctive ancient wheat species with specific protein and amino acid characteristics compared with polyploid wheat species [7,21]. However, the present results also show that such favourable characteristics should not be generalized to all ancient wheat materials, because the amino acid scores differed markedly among the analysed samples.

The role of breeding and selection should also be considered when interpreting differences between ancient and modern wheat materials. The previous study reported that breeding practices may modify the free amino acid composition of wholegrain flour, indicating that genetic selection can influence nitrogen metabolism and nutrient balance in wheat grain [25]. Although free amino acids are not directly equivalent to protein-bound amino acids determined after hydrolysis, these findings support the broader view that wheat improvement may affect amino acid-related nutritional traits. Similarly, comparative reviews emphasize that compositional differences between ancient and modern wheats contribute to nutritional variability, but that this variability is species- and material-dependent rather than uniform across all ancient wheats [6,26].

Overall, the amino acid analysis demonstrated that the analysed wheat materials differed not only in total protein content but also in protein quality. Selected ancient wheat materials, particularly emmer, Kamut, and einkorn, showed more favourable scores for some essential amino acids than common wheat and durum wheat. Nevertheless, lysine and histidine remained limiting across flour and pasta samples, confirming that higher protein content does not necessarily correspond to a balanced essential amino acid profile. These findings support the need to evaluate both quantitative and qualitative protein parameters when assessing the nutritional value of ancient and modern wheat-based products.

### 3.3. Gliadin Content in Flour and Pasta from Different Wheat Species

**Figure 2:**
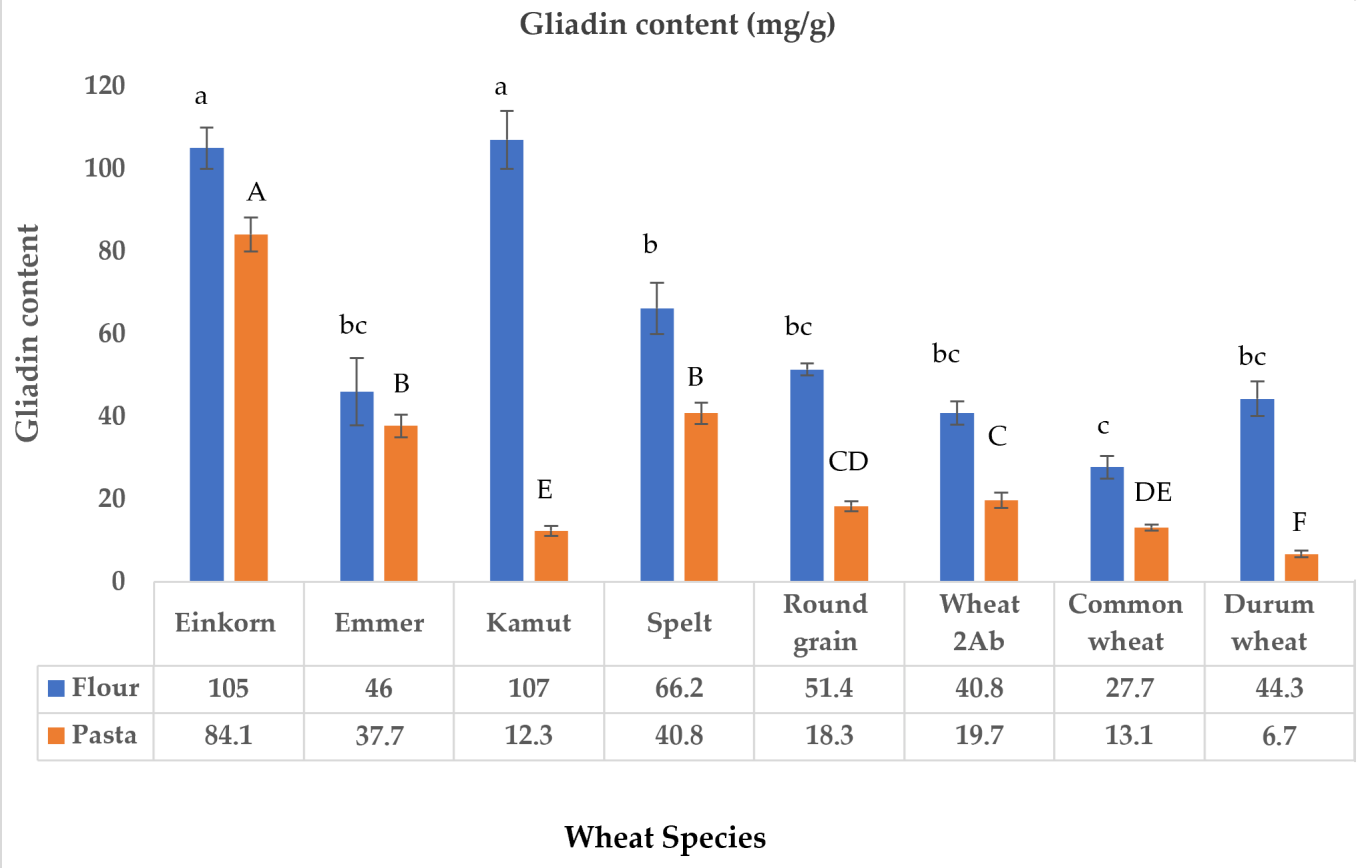
Gliadin content (mg g^−1^) in wheat flours and corresponding pasta products determined by direct ELISA using a polyclonal anti-gliadin antibody (Sigma A1052) under the applied assay conditions. Different lowercase letters indicate statistically significant differences among flour samples, whereas different uppercase letters indicate statistically significant differences among pasta samples according to Tukey’s HSD test (*p* < 0.05).

Gliadin content in flours and corresponding pasta products was quantified by direct ELISA using a polyclonal anti-gliadin antibody (Figure 2). Accordingly, the reported values should be understood as ELISA-based gliadin determinations obtained with the applied polyclonal antibody system. Across all analysed wheat materials, ELISA-detectable gliadin content was higher in flour than in the respective pasta samples, indicating that processing affected gliadin extractability and/or the accessibility of epitopes recognized by the analytical antibody.

Among the flours, einkorn exhibited the highest gliadin content (105 mg g^−1^), followed by Kamut, Spelt, and round grain wheat. In contrast, Common wheat and Durum wheat contained substantially lower amounts (27.7 and 29.4 mg g^−1^, respectively). These differences reflect inherent genetic variation in gluten protein composition among wheat species. Studies have reported that einkorn is characterized by a distinct gluten protein profile with elevated omega and alpha-gliadin fractions, which may influence both technological performance and immunogenic potential [27,28].

The high ELISA-detectable gliadin content observed in einkorn flour, especially in relation to its total protein content, may be interpreted in the context of wheat ploidy level and genome composition. Einkorn (*Triticum monococcum*) is a diploid wheat species with the AA genome, whereas durum wheat and Khorasan wheat/Kamut (*Triticum turgidum* ssp. *turanicum*) are tetraploid wheats with the AABB genome, and common wheat (*Triticum aestivum*) is a hexaploid wheat with the AABBDD genome. These differences in genome composition are associated with variation in gluten protein architecture, including the relative contribution of gliadin and glutenin fractions. In diploid and tetraploid wheats lacking the D genome, the gluten protein profile may be relatively more weighted toward monomeric gliadin fractions than in some hexaploid bread wheats, in which the D genome contributes important glutenin subunits involved in gluten network formation [7,8,9]. Therefore, the higher ELISA-detectable gliadin contents observed in einkorn and, to a lesser extent, in Kamut flour are consistent with literature reports describing ploidy- and genome-related differences in gluten protein composition. However, because direct genomic or proteomic profiling was not performed in the present study, these observations should be interpreted as material-related trends supported by known species-level differences, rather than as direct molecular characterization of the analysed samples. The lower ELISA-detectable gliadin levels observed in common wheat and durum wheat may also reflect species- and breeding-related differences in gluten protein composition, as modern wheat breeding has strongly focused on technological quality, including dough strength and processing performance [29,30].

Processing into pasta was associated with a reduction in ELISA-detectable gliadin content in all analysed wheat materials. Einkorn pasta retained the highest gliadin content (84.1 mg g^−1^), whereas Kamut pasta exhibited the greatest reduction after processing (nearly 9 times lower in comparison to flour). Common wheat and durum wheat pastas showed the lowest ELISA-detectable gliadin contents (13.1 and 6.7 mg g^−1^, respectively), more than twelve-fold lower than that observed in einkorn pasta.

The reduction in ELISA-detectable gliadin after pasta processing is consistent with structural and physicochemical changes occurring during hydration, mixing, extrusion, and drying. Protein denaturation, aggregation, and reduced extractability induced by thermal treatment can significantly influence antibody accessibility in ELISA-based quantification [31,32]. Heat-induced formation of disulfide-linked aggregates and enhanced protein–protein interactions may reduce gliadin solubility and limit epitope exposure, thereby decreasing measurable immunoreactivity without necessarily reducing total protein content [33]. Similar reductions in detectable gliadin after thermal processing have been reported in baked and heat-treated gluten systems, where structural rearrangements impair antibody binding rather than eliminate protein fractions [34,35].

Overall, these results show that gliadin detectability depends not only on the wheat material analysed, but also on the processing state of the product. Importantly, lower ELISA-detectable gliadin content in pasta should not be interpreted as direct evidence of reduced IgE-binding capacity, because processing may reduce gliadin extractability and alter the accessibility of epitopes recognized by the analytical anti-gliadin antibody. These changes do not necessarily correspond to changes in the accessibility of IgE-reactive epitopes, which were further evaluated by slot blot analysis using sera from allergic individuals.

### 3.4. Comparative IgE-Binding Analysis of Wheat Protein Extracts by Slot Blot

**Figure 3:**
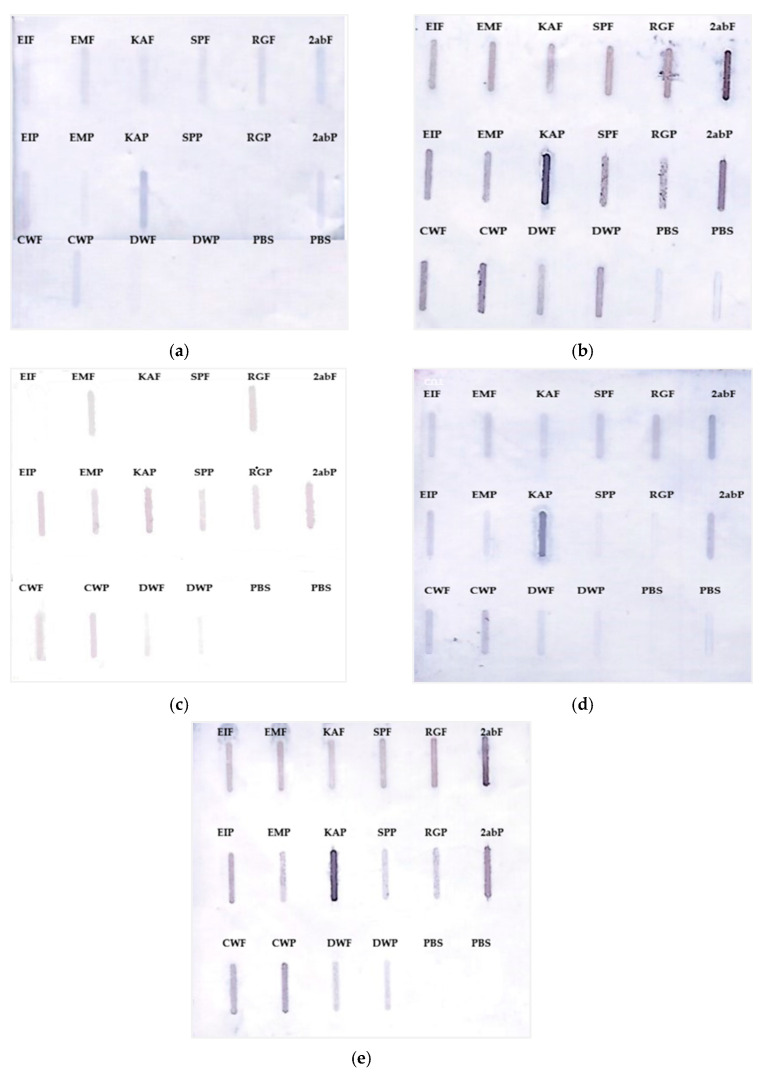
IgE-binding patterns of wheat flour and pasta protein extracts assessed by slot blot. Representative membranes show protein extracts from wheat flours and corresponding pasta products probed with sera from allergic individuals with different sensitization profiles: (**a**) Serum I; (**b**) Serum II; (**c**) Serum III; (**d**) Serum IV; and (**e**) Serum V. Detailed sensitization profiles of the sera are presented in Table 2. The observed signals represent IgE binding to components present in the tested wheat protein extracts.

The IgE-binding properties of wheat flour and pasta protein extracts were evaluated by slot blot analysis using five sera obtained from allergic individuals with different sensitization profiles. Representative slot blot membranes are presented in Figure 3. The assay was used as a comparative immunochemical approach to evaluate the relative IgE-binding capacity of the tested extracts and was not intended to identify individual wheat protein fractions. Accordingly, the observed signals were interpreted as relative immunoreactivity of the tested extracts, not as IgE binding to purified gliadin alone.

The results of densitometric analysis are provided in the Supplementary Materials (Tables S1–S5). It should be emphasized that individual membranes were probed with sera from different patients; therefore, signal intensities reflect serum-specific IgE recognition patterns. For this reason, densitometric analysis was performed separately for each membrane, and relative signal intensities should be compared only within the same membrane/serum, not directly between different membranes. The percentage values presented in Supplementary Tables S1–S5 therefore refer only to relative IgE-binding intensities within each membrane/serum and should not be used for direct comparison between different sera.

The IgE-binding patterns differed markedly depending on the serum used, wheat species, and sample form, i.e., flour or pasta. Overall, pasta extracts showed stronger and more clearly detectable IgE-binding signals than the corresponding flour extracts in several wheat species, particularly in analyses performed with Sera II, III, and V. However, the intensity and distribution of the signals were highly serum-dependent, indicating that IgE recognition was strongly influenced by the individual sensitization profile of each serum donor.

Serum I showed generally weak IgE-binding patterns, with several flour samples producing faint or undetectable signals. Among all samples analysed with this serum, Kamut pasta (KAP) showed the highest IgE-binding intensity and was therefore used as the reference sample (100%). Wheat 2Ab flour (2abF) exhibited comparatively strong binding, although its signal intensity was approximately 55% lower than that of KAP. Common wheat pasta (CWP) and round grain wheat flour (RGF) also showed moderate detectable signals, whereas Emmer pasta (EMP) showed the weakest IgE-binding response, reaching only approximately 7% of the KAP signal.

In contrast, Serum II produced broader and substantially stronger IgE-binding patterns across both flour and pasta extracts. Ancient wheat species, particularly Kamut, exhibited intense and clearly detectable signals, whereas modern wheat samples generally showed lower binding intensities. Among pasta samples, KAP again showed the highest IgE-binding intensity and was used as the reference sample (100%). Relative to KAP, Einkorn pasta (EIP), Emmer pasta (EMP), round grain wheat pasta (RGP), and Durum wheat pasta (DWP) showed approximately 54–60% lower IgE binding, whereas Spelt pasta (SPP) and Common wheat pasta (CWP) exhibited intermediate reductions of approximately 47% and 37%, respectively. Among flour extracts, wheat 2Ab flour (2abF) showed the strongest IgE-binding intensity, followed by round grain wheat flour (RGF), while the remaining wheat species displayed moderate or weaker binding patterns.

Serum III, characterized by strong grass pollen sensitization, produced moderate IgE-binding signals, particularly among ancient wheat flour extracts. Kamut pasta (KAP) again showed the highest IgE-binding intensity and was used as the reference sample (100%). Relative to KAP, Einkorn pasta (EIP) and round grain wheat flour (RGF) retained comparatively strong IgE binding, showing only approximately 13% lower signal intensity, whereas 2Ab pasta (2abP) showed a 16% reduction. Emmer flour (EMF) and Common wheat pasta (CWP) displayed intermediate IgE-binding intensities, while Durum wheat pasta (DWP) showed the weakest detectable signal, with approximately 80% lower IgE binding compared with KAP.

Serum IV produced weaker but clearly detectable IgE-binding patterns across most samples. Although this serum was characterized by strong cod sensitization, additional sensitization to soy and dog epithelium was also present; therefore, the observed signals should be interpreted cautiously and primarily as an individual IgE-recognition pattern rather than as evidence of a specific wheat-related sensitization. Similar to the results obtained with Sera II and III, KAP showed the strongest IgE-binding intensity. Flour extracts generally showed lower reactivity than the corresponding pasta samples, although round grain wheat flour (RGF) and wheat 2Ab flour (2abF) displayed comparatively stronger flour signals. Among pasta samples, Common wheat pasta (CWP) and round grain wheat pasta (RGP) demonstrated moderate IgE binding, whereas Emmer pasta (EMP), Spelt pasta (SPP), and 2Ab pasta (2abP) exhibited weak or undetectable signals.

Serum V, characterized by strong timothy grass sensitization and additional tree pollen sensitization, produced broader and more intense IgE-binding patterns across both flour and pasta extracts. Kamut pasta (KAP) again exhibited the highest IgE-binding intensity and was used as the reference sample (100%). Wheat 2Ab flour (2abF) retained relatively strong IgE binding, with signal intensity approximately 19% lower than KAP, followed by 2Ab pasta (2abP), which showed a 43% reduction. Common wheat pasta (CWP) and round grain wheat flour (RGF) also exhibited moderate detectable signals, whereas Spelt pasta (SPP) and Durum wheat pasta (DWP) showed substantially lower IgE-binding intensities.

In the present study, the strongest and broadest IgE-binding patterns were observed with Sera II and V, both characterized by pronounced sensitization to grass and/or tree pollens, whereas Sera I and IV produced weaker or more limited signals. In several membranes, pasta extracts showed stronger IgE-binding than the corresponding flour extracts, with Kamut pasta (KAP) repeatedly exhibiting the highest or one of the highest signal intensities. Taken together, these IgE-binding patterns indicate that processing from flour to pasta may modify the accessibility of IgE-reactive proteins or epitopes. Hydration, extrusion, drying, and cooking can alter protein conformation, promote aggregation, and reorganize the gluten network, thereby affecting epitope exposure and allergen recognition [15,36,37]. Thus, the higher signal intensity observed in Kamut pasta should not be interpreted simply as a higher amount of allergenic proteins, but rather as evidence that IgE-reactive components remained detectable after processing. Similar processing-related changes in allergenic protein detectability have been reported for wheat-based products [38].

The marked serum-dependent differences observed in the present study suggest that IgE recognition was strongly influenced by individual sensitization profiles. In particular, the broader IgE-binding patterns obtained with Sera II, III, and V may indicate that IgE from grass- and/or tree pollen-sensitized individuals recognized components present in the tested wheat protein extracts. This should not be interpreted as evidence of clinically confirmed pollen–food cross-reactivity, because detailed clinical information on symptoms after ingestion of wheat, pasta, or other cereal products was not available for all patients. However, IgE recognition of structurally related epitopes in plant-derived foods has been described in pollen-sensitized individuals and may involve conserved plant allergens, including profilins, cross-reactive carbohydrate determinants, or related epitopes [11,39,40,41]. Importantly, Kamut pasta consistently retained strong IgE-binding signals, indicating that processing did not eliminate IgE-reactive components in this material. Therefore, the improved protein-related nutritional characteristics of ancient wheat materials should not be interpreted as evidence of reduced IgE-binding capacity.

## 4. Conclusions

The present study provides an integrated analytical evaluation of protein quality and IgE-binding properties of flour and pasta products prepared from eight ancient and modern wheat materials used in industrial pasta production. The results showed that the analysed wheat materials differed substantially in total protein content, essential amino acid composition, ELISA-detectable gliadin content, and IgE-binding patterns. These differences were material-dependent and were further influenced by processing into pasta.

Spelt and Kamut showed the highest total protein contents among the analysed materials, while selected ancient wheat materials, particularly emmer, Kamut, and einkorn, exhibited more favourable scores for some essential amino acids than common wheat and durum wheat. However, lysine and histidine remained the main limiting amino acids across the analyzed samples. These results confirm that protein quality in wheat-based products cannot be assessed based on total protein content alone but requires consideration of essential amino acid composition.

Gliadin content also differed markedly among the analyzed materials. Einkorn flour showed the highest ELISA-detectable gliadin level, reflecting the specific gluten protein composition of this diploid wheat and the high contribution of monomeric gliadin fractions. Processing into pasta reduced ELISA-detectable gliadin levels in all analyzed wheat materials, indicating that industrial processing modifies protein extractability and the accessibility of antibody-recognized epitopes. However, the reduction in ELISA-detectable gliadin did not correspond to the elimination of IgE-binding activity.

Slot blot analysis demonstrated that IgE-binding patterns depended on the wheat material, product form, and individual serum profile. IgE-reactive components remained detectable in both flour and pasta extracts, and in several cases, pasta samples showed stronger or more clearly detectable IgE-binding signals than the corresponding flours. Kamut pasta repeatedly showed one of the strongest IgE-binding responses across different sera, indicating that processing did not eliminate IgE-reactive components in this material.

Overall, the findings show that ancient and modern wheat materials differ not only in protein-related nutritional characteristics, but also in gliadin detectability and IgE-binding capacity. Selected ancient wheat materials may offer favourable nutritional features; however, these features should not be interpreted as evidence of reduced IgE-binding potential. Therefore, assessment of wheat-based products, particularly those produced from diverse ancient and modern wheat materials, should combine protein quality evaluation with analysis of gliadin detectability and IgE-binding properties. This approach provides a more complete understanding of how wheat material selection and pasta processing shape the final nutritional and immunological profile of cereal products.

## Figures and Tables

**Table 1:** Basic characteristics of flour and pasta samples and their codes.

Wheat Species	Flour Sample Code	Dry Matter (%)	Pasta Sample Code	Dry Matter (%)
Common wheat (*Triticum aestivum*)	CWF	89.5	CWP	90.3
Durum wheat (*Triticum durum*)	DWF	89.2	DWP	90.6
Round grain wheat	RGF	89.4	RWP	90.4
Einkorn (*Tritticum monococcum*)	EIF	88.6	EIP	90.3
Emmer (*Triticum dicoccum*)	EMF	88.9	EMP	90.7
Kamut (*Triticum turgidum* ssp. *turanicum*)	KAF	88.1	KAP	90.4
Spelt (*Triticum spelta*)	SPF	88.0	SPP	90.8
2Ab wheat	2abF	89.1	2abP	90.6

**Table 2:** Specific IgE sensitization profiles of patients’ sera.

Serum	Allergen/Source	Allergen/Source Category	Class
**I**	Timothy meadowgrass Birch and oak pollen Common mugwort Peanuts Hazelnuts Carrots Apple	Inhalant/pollen allergen Inhalant/pollen allergen Inhalant/pollen allergen Food allergen Food allergen Food allergen Food allergen	4 4 2 3 2 3 4
**II**	Birch and oak pollen Grey alder pollen Hazel pollen Timothy meadowgrass Rye pollen Wheat flour	Inhalant/pollen allergen Inhalant/pollen allergen Inhalant/pollen allergen Inhalant/pollen allergen Inhalant/pollen allergen Food allergen/flour allergen	6 6 6 6 4 3
**III**	6 grass mix Banana Pork Beef Flour mix Birch and oak pollen Pollens of alder and hazel	Inhalant/pollen allergen Food allergen Food allergen Food allergen Food allergen/flour allergen Inhalant/pollen allergen Inhalant/pollen allergen	6 3 2 2 3 3 4
**IV**	Cod Soy Dog epidermis	Food allergen Food allergen Animal-derived inhalant allergen	6 2 2
**V**	Birch pollen Grey alder pollen Hazel pollen Oak pollen Timothy meadowgrass Rye pollen Mugwort pollen Horse and dog epidermis	Inhalant/pollen allergen Inhalant/pollen allergen Inhalant/pollen allergen Inhalant/pollen allergen Inhalant/pollen allergen Inhalant/pollen allergen Inhalant/pollen allergen Animal-derived inhalant allergen	3 3 2 3 6 2 2 2

Note: The table presents specific IgE sensitization profiles determined using Polycheck^®^ diagnostic panels. Listed items refer to allergens or allergen sources included in the diagnostic panels and do not indicate the route of sensitization or clinically confirmed allergy. The allergen/source category column was added to distinguish inhalant/pollen allergens, food allergens, food/flour allergens, and animal-derived inhalant allergens.

**Table 3:** Essential amino acid compositions (mg/g protein) and nutritional score values. (% FAO/WHO requirement pattern) of raw flour from eight distinct wheat varieties.

Wheat Material Flour Form	Histidine	Isoleucine	Leucine	Lysine	Aromatic AA	Threonine	Valine
	(His)	(Ile)	(Leu)	(Lys)	(AAA)	(Thr)	(Val)
Enikorn	0.5 ± 0.1 ^ab^ (3.12%)	4.3 ± 1.1 ^a^ (14.33%)	8.9 ± 2.4 ^bc^ (14.59%)	11.4 ± 16.1 ^b^ (23.75%)	11.7 ± 4.6 ^ab^ (28.53%)	4.6 ± 1.1 ^ab^ (18.40%)	5.2 ± 1.2 ^ab^ (13.00%)
Emmer	0.7 ± 0.05 ^a^ (4.37%)	5.3 ± 0.0 ^a^ (17.66%)	11.3 ± 0.3 ^a^ (18.25%)	30.0 ± 0.0 ^a^ (62.50%)	12.1 ± 1.0 ^a^ (29.51%)	5.6 ± 0.3 ^ab^ (22.40%)	6.6 ± 0.1 ^a^ (16.50%)
Kamut	0.8 ± 0.2 ^a^ (5.00%)	4.8 ± 0.1 ^a^ (16.00%)	11.0 ± 0.3 ^ab^ (18.00%)	2.2 ± 0.0 ^c^ (4.58%)	10.2 ± 0.2 ^ab^ (24.87%)	9.5 ± 5.0 ^a^ (38.00%)	5.9 ± 0.2 ^ab^ (14.75%)
Spelt	0.7 ± 0.05 ^a^ (4.37%)	4.8 ± 0.1 ^a^ (16.00%)	10.8 ± 0.1 ^ab^ (17.70%)	2.2 ± 0.1 ^c^ (4.58%)	10.2 ± 0.2 ^ab^ (24.87%)	4.6 ± 0.1 ^ab^ (18.40%)	6.0 ± 0.1 ^ab^ (15.00%)
Ground Grain	0.5 ± 0.1 ^ab^ (3.12%)	6.2 ± 3.1 ^a^ (20.66%)	6.7 ± 3.6 ^bc^ (10.98%)	1.8 ± 0.3 ^c^ (3.75%)	7.6 ± 1.2 ^b^ (18.53%)	3.6 ± 0.5 ^b^ (14.40%)	4.4 ± 0.5 ^bc^ (11.00%)
Wheat 2Ab	1.0 ± 0.25 ^ab^ (6.25%)	4.7 ± 2.4 ^a^ (15.66%)	6.7 ± 2.9 ^bc^ (10.98%)	1.5 ± 0.8 ^c^ (3.12%)	9.4 ± 4.1 ^ab^ (22.92%)	4.5 ± 1.2 ^ab^ (18.00%)	5.4 ± 2.9 ^ab^ (13.75%)
Common wheat	0.3 ± 0.05 ^b^ (1.87%)	2.2 ± 0.3 ^a^ (7.30%)	5.2 ± 0.5 ^c^ (8.52%)	1.0 ± 0.1 ^c^ (2.08%)	5.0 ± 0.4 ^c^ (12.19%)	2.2 ± 0.1 ^b^ (8.80%)	2.6 ± 0.2 ^c^ (6.50%)
Durum wheat	0.3 ± 0.1 ^b^ (1.87%)	2.4 ± 0.4 ^a^ (8.00%)	5.9 ± 0.8 ^c^ (9.67%)	0.9 ± 0.0 ^c^ (1.87%)	5.5 ± 0.5 ^c^ (13.41%)	2.4 ± 0.0 ^b^ (9.60%)	3.0 ± 0.4 ^c^ (7.50%)
FAO/WHO Reference pattern	16	30	61	48	41	25	40

Note. Values are expressed as mean ± standard deviation (SD) of three replicates and are given in mg/g protein. Values in parentheses indicate amino acid scores calculated as percentages of the FAO/WHO (2013) reference pattern for older children, adolescents, and adults. Different lowercase superscript letters within the same column indicate statistically significant differences at *p* < 0.05. AAA, aromatic amino acids, calculated as the sum of phenylalanine and tyrosine. Sulfur amino acids (SAA) and tryptophan (Trp) were not included in the evaluation.

**Table 4:** Essential amino acid compositions (mg/g protein) and nutritional score values (% FAO/WHO requirement pattern) of pasta products from eight distinct wheat varieties.

Wheat Material Flour Form	Histidine	Isoleucine	Leucine	Lysine	Aromatic AA	Threonine	Valine
	(His)	(Ile)	(Leu)	(Lys)	(AAA)	(Thr)	(Val)
Enikorn	1.8 ± 0.2 ^a^ (11.25%)	13.9 ± 1.0 ^a^ (46.33%)	29.7 ± 1.6 ^b^ (48.68%)	5.8 ± 0.3 ^a^ (12.08%)	26.1 ± 3.6 ^a^ (63.00%)	13.4 ± 0.7 ^ab^ (5.36%)	16.6 ± 1.0 ^b^ (41.50%)
Emmer	0.87 ± 0.1 ^bc^ (5.43%)	14.8 ± 8.4 ^a^ (49.33%)	18.7 ± 5.0 ^cd^ (30.65%)	11.6 ± 8.9 ^a^ (24.16%)	26.9 ± 10.6 ^a^ (65.00%)	6.7 ± 0.5 ^cd^ (26.80%)	13.0 ± 4.9 ^bc^ (32.50%)
Kamut	0.8 ± 0.3 ^bc^ (5.00%)	16.7 ± 0 ^a^ (55.00%)	19.4 ± 0.1 ^cd^ (31.80%)	17.0 ± 0.1 ^a^ (35.41%)	40.6 ± 0.2 ^a^ (99.20%)	5.9 ± 2.2 ^cd^ (23.60%)	14.7 ± 0.0 ^bc^ (36.75%)
Spelt	1.2 ± 0.7 ^abc^ (7.50%)	11.9 ± 7.3 ^a^ (39.66%)	14.8 ± 4.8 ^de^ (24.26%)	12.6 ± 3.9 ^a^ (26.25%)	27.8 ± 19.0 ^a^ (67.80%)	8.7 ± 4.6 ^bcd^ (34.80%)	10.2 ± 4.5 ^bc^ (25.50%)
Ground Grain	0.6 ± 0.5 ^c^ (3.75%)	4.03 ± 1.4 ^a^ (13.43%)	10.8 ± 1.3 ^e^ (17.70%)	1.17 ± 0.06 ^a^ (2.43%)	10.1 ± 0.9 ^a^ (24.39%)	4.2 ± 0.8 ^d^ (16.80%)	5.0 ± 1.6 ^c^ (12.50%)
Wheat 2Ab	1.4 ± 0.20 ^ab^ (8.75%)	17.9 ± 5.0 ^a^ (59.66%)	25.1 ± 2.7 ^bc^ (41.14%)	11.93 ± 8.0 ^a^ (24.85%)	40.5 ± 15.1 ^a^ (98.78%)	10.2 ± 0.5 ^bc^ (40.80%)	16.4 ± 1.1 ^b^ (41.00%)
Common wheat	0.6 ± 0.2 ^c^ (3.75%)	16.0 ± 10.5 ^a^ (53.33%)	19.6 ± 7.30 ^cd^ (32.13%)	12.2 ± 11.1 ^a^ (25.41%)	38.1 ± 26.3 ^a^ (92.92%)	4.3 ± 0.8 ^d^ (17.20%)	13.27 ± 7.2 ^bc^ (33.17%)
Durum Wheat	2.0 ± 0.0 ^a^ (12.50%)	17.9 ± 0.1 ^a^ (59.66%)	38.6 ± 0.1 ^a^ (63.31%)	5.9 ± 0.0 ^a^ (12.29%)	35.2 ± 0.2 ^a^ (85.00%)	14.4 ± 0.0 ^a^ (57.60%)	20.8 ± 0.1 ^a^ (52.00%)
**FAO/WHO** **Reference** **pattern**	16	30	61	48	41	25	40

Note. Values represent mean analytical concentrations ± standard deviation (SD) of three separate replicates, expressed uniformly in mg/g protein. Numbers in parentheses indicate the corresponding amino acid scores calculated relative to the FAO/WHO reference baseline target. Different lowercase superscript letters within the same column indicate statistically significant differences at *p* < 0.05. AAA = Aromatic Amino Acids (Phenylalanine + Tyrosine). Sulfur Amino Acids (SAA) and Tryptophan (Trp) were excluded from evaluation.

## Data Availability

The original contributions presented in the study are included in the article/Supplementary Material, further inquiries can be directed to the corresponding author.

## Supplementary Materials

The following supporting information can be downloaded at: https://www.mdpi.com/article/10.3390/foods15152605/s1, Table S1: Densitometric analysis of IgE-binding intensities of wheat flour and pasta protein extracts using Serum I; Table S2: Densitometric analysis of IgE-binding intensities of wheat flour and pasta protein extracts using Serum II; Table S3: Densitometric analysis of IgE-binding intensities of wheat flour and pasta protein extracts using Serum III; Table S4: Densitometric analysis of IgE-binding intensities of wheat flour and pasta protein extracts using Serum IV; Table S5: Densitometric analysis of IgE-binding intensities of wheat flour and pasta protein extracts using Serum V.

## Abbreviations

The following abbreviations are used in this manuscript:

ELISA	Enzyme-Linked Immunosorbent Assay
IgE	Immunoglobulin E
UHPLC	Ultra -High-Performance Liquid chromatography
TBS	Tris-Buffered Saline
BSA	Bovine serum albumin
PVDF	Polyvinylidene Fluoride
