# Supplementary material for: Protein Quality and IgE-Binding in Ancient and Modern Wheat: An Analytical Study
Source: Foods. 2026 Jul 25;15(15):2605. doi: 10.3390/foods15152605 (PMC13465718; doi:10.3390/foods15152605)
Supplement: Supplementary file 1 [file foods-15-02605-s001.zip › foods-4416437-supplementary.pdf]

Table S1. Densitometric analysis of IgE-binding intensities of wheat flour and pasta protein extracts using Serum I.

Label	Area	Integrated density [a.u]	Corrected integrated intensity [a.u]	Percentage of intensity [%] *
EIF	804	15121	2659	13
EMF	804	19069	6607	31
KAF	804	16744	4282	20
SPF	804	18026	5564	26
RGF	804	21072	8610	41
2abF	804	24151	11689	55
CWF	804	No band	-	-
DWF	804	No band	-	-
EIP	804	27156	14694	70
EMP	804	13971	1509	7
KAP	804	33547	21085	100
SPP	804	No Band	-	-
RGP	804	No Band	-	-
2abP	804	20392	7930	38
CWP	804	21545	9083	43
DWP	804	No band	-	-
PBS-1	804	No band	-	-
PBS-2	804	No band	-	-

*Note Percentage values are relative values calculated for this serum by setting the strongest signal as 100%. They should not be used for direct quantitative comparison with values obtained for other sera. "No band" indicates that the signal in the respective region of interest was not distinguishable from the local membrane background after background subtraction.

Table S2. Densitometric analysis of IgE-binding intensities of wheat flour and pasta protein extracts using Serum II.

Label	Area	Integrated density [a.u]	Corrected integrated intensity [a.u]	Percentage of intensity [%]*
EIF	804	44784	44224	35
EMF	804	54357	53677	42
KAF	804	45040	44480	35
SPF	804	54529	53849	42
RGF	804	66310	65490	82
2abF	804	125522	123962	97
CWF	804	69763	68893	54
DWF	804	43382	42842	34
EIP	804	59634	58894	46
EMP	804	51243	50603	40
KAP	804	128958	127358	100
SPP	804	67864	67024	53
RGP	804	59912	59162	46
2abP	804	91278	90138	71
CWP	804	81225	80215	63
DWP	804	59193	58453	46
PBS-1	804	No band	-	-
PBS-2	804	No band	-	-

*Note Percentage values are relative values calculated for this serum by setting the strongest signal as 100%. They should not be used for direct quantitative comparison with values obtained for other sera. "No band" indicates that the signal in the respective region of interest was not distinguishable from the local membrane background after background subtraction.

Table S3. Densitometric analysis of IgE-binding intensities of wheat flour and pasta protein extracts using Serum III.

Label	Area	Integrated density [a.u]	Corrected integrated intensity [a.u]	Percentage of intensity [%]*
EIF	371	No band	-	-
EMF	371	18796	18796	74
KAF	371	No band	-	-
SPF	371	No band	-	-
RGF	371	22293	22293	87
2abF	371	No band	-	-
CWF	371	20546	20546	80
DWF	371	8753	8753	34
EIP	371	22350	22350	87
EMP	371	19818	19818	78
KAP	371	25565	25565	100
SPP	371	14483	14483	57
RGP	371	14949	14949	58
2abP	371	21586	21586	84
CWP	371	18725	18725	73
DWP	371	5230	5230	20
PBS-1	371	No band	-	-
PBS-2	371	No band	-	-

*Note Percentage values are relative values calculated for this serum by setting the strongest signal as 100%. They should not be used for direct quantitative comparison with values obtained for other sera. "No band" indicates that the signal in the respective region of interest was not distinguishable from the local membrane background after background subtraction.

Table S4. Densitometric analysis of IgE-binding intensities of wheat flour and pasta protein extracts using Serum IV.

Label	Area	Integrated density [a.u]	Corrected integrated intensity [a.u]	Percentage of intensity [%] *
EIF	371	14001	4911.5	19
EMF	371	15333	6243.5	24
KAF	371	13921	4831.5	18
SPF	371	15601	6511.5	25
RGF	371	23551	14461.5	55
2abF	371	19316	10226.5	39
CWF	371	17072	7982.5	30
DWF	371	12502	3412.5	13
EIP	371	13618	4528.5	17
EMP	371	No band	No band	-
KAP	371	35323	26233.5	100
SPP	371	No band	-	-
RGP	371	18350	9260.5	35
2abP	371	No band	-	-
CWP	371	19614	10524.5	40
DWP	371	9678	588.5	2
PBS-1	371	No band	-	-
PBS-2	371	No band	-	-

*Note Percentage values are relative values calculated for this serum by setting the strongest signal as 100%. They should not be used for direct quantitative comparison with values obtained for other sera. "No band" indicates that the signal in the respective region of interest was not distinguishable from the local membrane background after background subtraction.

Table S5: Densitometric analysis of IgE-binding intensities of wheat flour and pasta protein extracts using Serum V.

Label	Area	Integrated density [a.u]	Corrected integrated intensity [a.u]	Percentage of intensity [%] *
EIF	371	16101	15174	28
EMF	371	16248	15321	28
KAF	371	13487	12560	23
SPF	371	18641	17714	32
RGF	371	24272	23345	42
2abF	371	45553	44626	81
CWF	371	20964	20037	36
DWF	371	10116	9189	17
EIP	371	21784	20857	38
EMP	371	13141	12214	22
KAP	371	55989	55062	100
SPP	371	10454	9527	17
RGP	371	11885	10958	20
2abP	371	32458	31531	57
CWP	371	24809	23882	43
DWP	371	7898	6971	13
PBS-1	371	No Band	-	-
PBS-2	371	No Band	-	-

Note:*Note Percentage values are relative values calculated for this serum by setting the strongest signal as 100%. They should not be used for direct quantitative comparison with values obtained for other sera. "No band" indicates that the signal in the respective region of interest was not distinguishable from the local membrane background after background subtraction.
